# Enhancing flood risk mitigation by advanced data-driven approach

**DOI:** 10.1016/j.heliyon.2024.e37758

**Published:** 2024-09-14

**Authors:** Ali S. Chafjiri, Mohammad Gheibi, Benyamin Chahkandi, Hamid Eghbalian, Stanislaw Waclawek, Amir M. Fathollahi-Fard, Kourosh Behzadian

**Affiliations:** a School of Civil Engineering, University of Tehran, Tehran, Iran; b Institute for Nanomaterials, Advanced Technologies and Innovation, Technical University of Liberec, Studentská 1402/2, 461 17, Liberec, Czech Republic; c Department of Civil and Environmental Engineering, Amirkabir University of Technology, Tehran, 1591634311, Iran; d Département d’Analytique, Opérations et Technologies de l’Information, Université de Québec à Montreal, 315, Sainte-Catherine Street East, H2X 3X2, Montreal, Canada; e New Era and Development in Civil Engineering Research Group, Scientific Research Center, Al-Ayen University, Nasiriyah, Thi-Qar, 64001, Iraq; f School of Computing and Engineering, University of West London, London, W5 5RF, UK; g Department of Civil, Environmental and Geomatic Engineering, University College London, Gower St, London, WC1E 6BT, UK; h Faculty of Civil and Environmental Engineering, Gdansk University of Technology, Narutowicza Street 11/12, 80-233, Gdansk, Poland

**Keywords:** Flood, Risk assessment, Data-driven, Machine learning, Sefidrud river

## Abstract

Flood events in the Sefidrud River basin have historically caused significant damage to infrastructure, agriculture, and human settlements, highlighting the urgent need for improved flood prediction capabilities. Traditional hydrological models have shown limitations in capturing the complex, non-linear relationships inherent in flood dynamics. This study addresses these challenges by leveraging advanced machine learning techniques to develop more accurate and reliable flood estimation models for the region. The study applied Random Forest (RF), Bagging, SMOreg, Multilayer Perceptron (MLP), and Adaptive Neuro-Fuzzy Inference System (ANFIS) models using historical hydrological data spanning 50 years. The methods involved splitting the data into training (50–70 %) and validation sets, processed using WEKA 3.9 software. The evaluation revealed that the nonlinear ensemble RF model achieved the highest accuracy with a correlation of 0.868 and an root mean squared error (RMSE) of 0.104. Both RF and MLP significantly outperformed the linear SMOreg approach, demonstrating the suitability of modern machine learning techniques. Additionally, the ANFIS model achieved an exceptional R-squared accuracy of 0.99. The findings underscore the potential of data-driven models for accurate flood estimating, providing a valuable benchmark for algorithm selection in flood risk management.

## Highlights

• Implementation of soft-sensor for forecasting flood disaster by the using different machine learning methods.
• Analysing the optimum condition for flood management by the response surface methodology.
• Data gathering of river flow with the application of statistical evaluations in the SefidRud catchment.
• Execution of a comprehensive decision support system for managing the river flow due to reduction of flood damages.

## Graphical abstract

**Figure:**
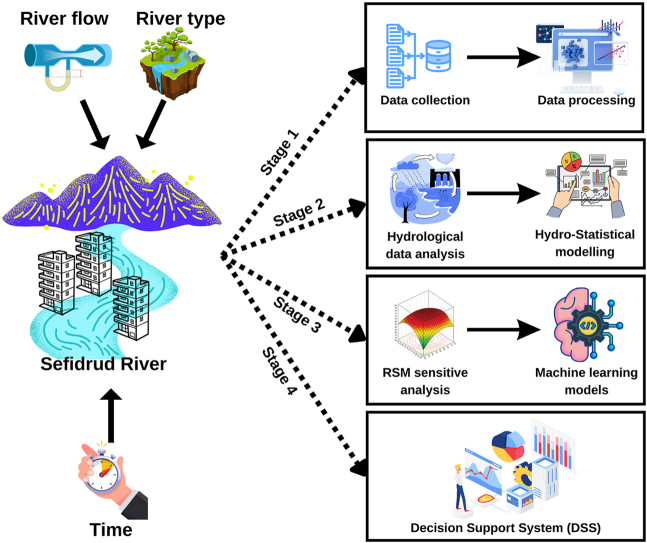

## 1 Introduction

Flashflood is a frequently observed disaster, which is taking its toll both in terms of loss of lives and economically [1]. Flash floods pose a considerable risk as they occur unexpectedly without a prior warning. Due to economic conditions and increased accessibility to water, individuals are relocating to floodplains. Consequently, these high-risk flood-prone areas have experienced growth since the 1950s [2]. According to the 2019 Asia-Pacific Disaster report by the Economic and Social Commission for Asia and the Pacific (ESCAP), Iran experienced annualized economic losses from disasters totaling around US$16.47 billion, constituting approximately 3.7 percent of its GDP. Notably, the unprecedented flood in 2019 impacted nearly 10 million people and caused a substantial economic toll of $4.27 billion. With ESCAP projecting a rise in flood losses by 2030, the management and impact of floods are increasingly becoming a significant concern within the risk landscape of the country [3].

Floods are among the most pervasive disasters, affecting millions of people worldwide annually. In Iran, the damage caused by floods is considerable and has surged at the end of the last decade. Alone, it claimed a significant share of the GDP in 2019 and 2020. Approximately 420,000 km^2^ of watersheds in Iran are at a moderate to high risk of flooding [4]. Approaches for flood protection can be categorized into two main types: 1) structural, which involves the construction of flood protection structures, and 2) non-structural measures, such as land-use planning, flood zoning, and flood alarming systems. Flood management through the structural approach lacks efficacy, primarily due to climate and land-use changes. Therefore, it needs to be complemented with alternative methods [5,6]. Therefore, flood management constitutes a multifaceted challenge that necessitates a robust decision-making platform.

An effective approach involves the use of a Decision Support System (DSS) comprising monitoring, prediction, and control components, facilitating intelligent, agile, and resilient management of such intricate issues [7]. The advent and evolution of DSSs tailored for flood-related scenarios reflect the strategy employed by policymakers in addressing complex challenges [8].

DSS is a category of information systems that enhances the decision-making process. In the context of floods, a DSS relies on real-time data from gauging stations, such as water levels and discharge at specific points. Using a model derived from historical station data, the DSS creates a soft sensor to issue alarms for imminent floods, enabling authorities to take necessary precautionary measures [9]. In this regard, Akbarian et al. [10] designed a DSS and implemented for real-time monitoring, prediction, and control of flood disaster management. This DSS integrated hydrological data mining, Machine Learning (ML), and Multi-Criteria Decision Making (MCDM) to create smart alarm and prevention systems. The DSS facilitated conditional management of floods across different clusters of climates in Iran by utilizing techniques such as KMeans clustering to determine hydrological homogeneity and ML algorithms like Nearest Neighbors Classification (NNC), Stochastic Gradient Descent (SGD), Gaussian Process Classifier (GPC), and Neural Network (NN) to evaluate the relationship between rainfall and flood disasters. In a recent study proposed by Zabihi et al. [7] in 2023, a DSS was developed to manage flood disasters effectively. Flood records from five provinces in Iran were clustered, and ML techniques such as Logistic Regression, NN, and Support Vector Machine (SVM) were employed to estimate flood disasters based on rainfall in different climates.

Flood Estimation (FE) models play a vital role in evaluating and managing risks associated with floods. These models are utilised by considering runoff production processes, ensuring data sufficiency, and taking into account catchment characteristics [11]. Due to the high uncertainties and challenges imposed by the traditional modellings, ML approaches are getting more and more popular for flood estimation, forecasting and management [12]. For example; frequent floods in Odisha, India, cause significant disruption and economic loss. Traditional forecasting is complex. Machine learning models, using various meteorological data, offer better accuracy. Random forest outperforms other models like Naïve Bayes and SVM in predicting floods [13]. By scrutinizing inputs from sensors and satellite imagery, ML models enable close-to-real-time flood monitoring, thereby facilitating swift identification and hastening response actions [14]. Through the intricate analysis of diverse factors such as land use, geography, and infrastructure, ML models proficiently gauge flood risk, aiding in pinpointing flood-prone regions and informing strategic management decisions [15]. Moreover, ML models streamline the evaluation of flood damage by harnessing satellite images and assorted data sources, thereby expediting decision-making processes for relief and recovery initiatives [16]. These models effectively simulate the complexity and unpredictability of natural processes, as evidenced by the works of [9,15].

ML models have exhibited a desirable level of robustness in FE process [17,18] and have demonstrated greater accuracy compared to traditional laborious methods [19]. Recent literature demonstrates that ML techniques hold significant potential for riverine flood forecasting in contrast to conventional methods [20]. Utilizing data-driven methodologies proves effective in capturing intricate nonlinear relationships and interactions among diverse hydrometeorological variables that impact the incidence of floods [21].

Ren et al. [22] in 2019 used two different algorithms (nearest neighbor and fireworks) to improve and create a hybrid flood estimation model. Their research focused on flash floods in small and medium catchments (case study Loess region) for three different subdivision of surface condition and flood grades. In the same year, Zahura et al. [23] applied ML method of Random Forest (RF) as an outcome model combined with physical one to obtain a high-resolution street-scale flood estimation in Virginia, USA. In 2021, Zarei et al. [24] developed an inflow estimation model using different ML algorithms such as SVM and ANN, Regression tree (RT) and genetic programming (GP) for the sake of optimum reservoir management.

In 2022, Khosravi et al. [12] introduced a CNN-BAT model for daily streamflow prediction, tested against MLP-BAT, ANFIS-BAT, SVR-BAT, and RF-BAT models using data from the Korkorsar catchment in northern Iran. The CNN-BAT model, leveraging 15 years of rainfall and streamflow data, demonstrated superior performance in various evaluation metrics, proving more effective than other algorithms. The integration of Rt, Rt-1, and Qt-1 as input variables was optimal, with the CNN-BAT model showing statistically significant improvements. In the same year, Zhou et al. [25] used an integral ML model along with a conceptual hydrological model to minimise error propagation in flood estimation. In 2023, Piadeh et al. [26] applied ML for flood prediction by developing a novel event-based decision support algorithm. ML techniques were employed in event-based data identification and generation stages, where ML models were trained to recognize and classify flood-related events. These events were then used to generate event-based datasets for flood forecasting.

More recently in 2024, Dtissibe et al. [27] developed and compared ML and DL models for short-term and long-term flood forecasting in the Far-North region of Cameroon. They collected temperature and rainfall time series data from the region and designed models including one-dimensional Convolutional Neural Networks (1D-CNN), Long Short-Term Memory networks (LSTM), and Multi-Layer Perceptrons (MLP). Defontaine et al. [28] aimed to improve flood prediction for the Garonne River in Toulouse by developing fully data-driven ML models. They used discharge and rainfall data from 36 flood events. They trained three models, i.e., linear regression, gradient boosting regressor, and MultiLayer Perceptron (MLP), to forecast discharge at 6-h and 8-h lead times.

This research addresses significant gaps in current flood management practices by integrating machine learning computations, statistical optimization techniques, and decision support system for river flow management. While ML has shown promise in flood estimation, its simultaneous integration with statistical optimization and DSS approaches remains largely unexplored. The study aims to develop comprehensive data-driven models to better address the complexities of flood management. Additionally, it explores the implementation of soft-sensor techniques for estimating flood disasters, which has been limited in previous research. The research also seeks to analyze the optimal conditions for flood management through response surface methodology, addressing a critical gap in current practices that often lack systematic analyses of mitigation strategies. Furthermore, the study aims to enhance data gathering and statistical evaluation practices in river catchments, particularly in the SefidRud catchment area, to improve the accuracy and reliability of flood estimating models. Lastly, it aims to develop a comprehensive DSS tailored for river flow management to provide decision-makers with valuable tools for enhancing flood risk mitigation and disaster management efforts, addressing the current lack of effective flood management practices in many regions.

The novelty of this research lies in its comprehensive and multi-faceted approach to flood risk mitigation. By leveraging a diverse set of advanced ML models, utilizing extensive historical data, integrating these models into a robust DSS, applying response surface methodology (RSM), and aligning with SDGs, this study provides significant advancements in the field of flood estimation and management. These innovations collectively offer a powerful framework for improving flood risk mitigation strategies in the Sefidrud River basin and potentially in other flood-prone regions globally.

The present research aims to develop comprehensive and optimized data-driven flood management models integrating machine learning computations and statistical optimization techniques for river flow management, explore the implementation of soft-sensor techniques, including Adaptive Neuro-Fuzzy Inference System (ANFIS), RF, MLP, Bagging, and SMOreg, for estimating flood disasters and enhancing the precision of flood estimation, analyze the optimal flow for flood management through the application of response surface methodology, and developing a comprehensive DSS tailored for river flow management, providing decision-makers with valuable tools for enhancing flood risk mitigation and disaster management efforts. Therefore, the key research highlights include:

• Implementation of soft-sensor for estimating flood disaster by the using ANFIS, RF, MLP, Bagging and SMOreg.
• Analyzing the optimum condition for flood management by the application of response surface methodology.
• Data gathering of river flow with the application of statistical evaluations in the SefidRud catchment.
• Data validation, calibration, and verification in the case study during 51 years.
• Execution of a complete DSS for managing the river flow due to reduction of flood damages.

In the following, materials and methods, including statistical data assessment, data gathering, case study, numerical and hydrological methods, ML computations and decision-making systems are presented in Section 2. Then, the main outcomes and outputs of the present research with focusing on comparison to other studies, are argued in Section 3. In Section 4, the main achievements with concentration on Sustainable Development Goals (SDGs) are appraised to express the most important findings and suggestions for future research.

## 2 Materials and methods

The study employs a data-driven modeling approach, utilizing machine learning techniques to formulate flood prediction models based on extensive historical hydrometric data. It evaluates both traditional hydrologic methods and contemporary machine learning algorithms, such as RF, MLP, Bagging, and SMOreg, to estimate flood events using historical data. In addition to these data-driven models, a process-based response surface methodology is applied to analyze the river flow data, providing insights into the interactions among tributary rivers. The machine learning models undergo training on 50–70 % of the available data and are subsequently tested on the remaining data to validate their accuracy. The overall model performance is assessed through various metrics, including correlation, root mean squared error, and relative error, in comparison to the requirements of flood estimation. Additionally, the study includes a comprehensive statistical analysis and flood frequency analysis of multi-decadal observed river flow data to characterize flood occurrence patterns in the case study. The aforementioned methodology is implemented in this particular case to enhance understanding and prediction of flood events.

**Fig. 1:**
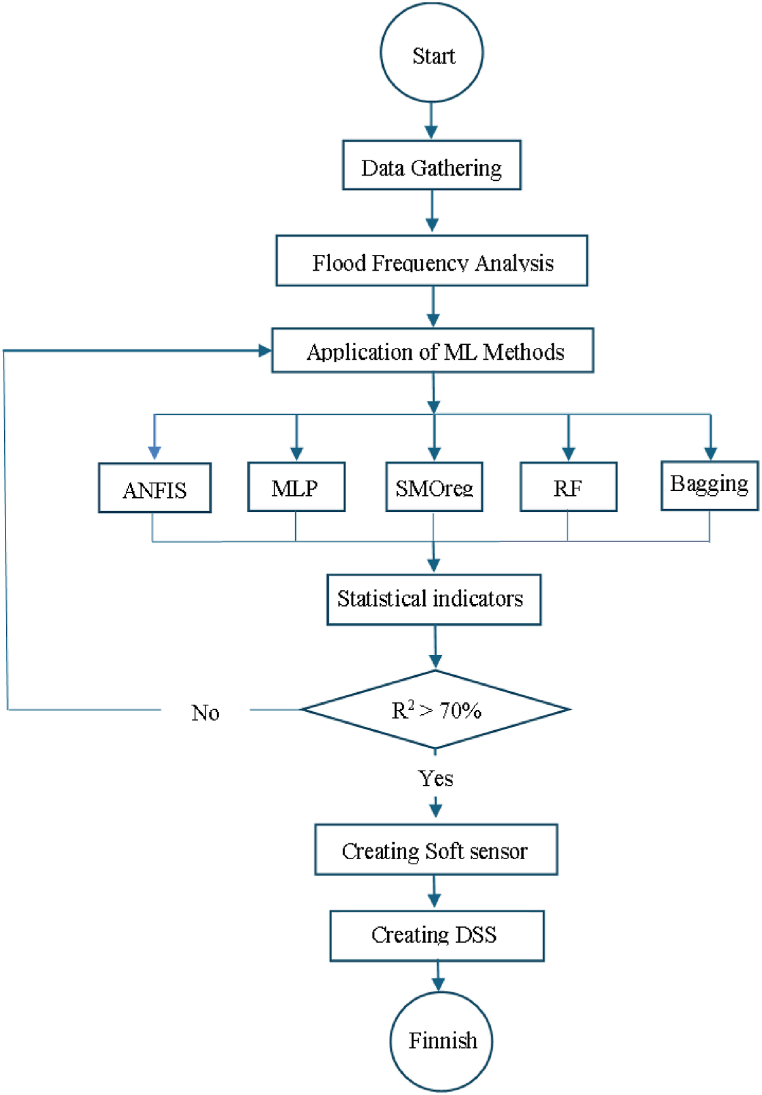
Workflow block diagram of the research.

In the present section, all numerical and simulation steps are appraised, and also the details of case study plus artificial intelligence details are conveyed. Most of the following data on the climatological, topographical and hydrological characteristics of the SefidRud has been provided in comprehensive water resource management studies conducted by Ref. [29]. The workflow of the research steps is presented as per Fig. 1.

### 2.1 Response surface methodology (RSM)

Researchers can forecast interactions that are difficult to run using response surface methods, which also provides them an indication of the response curvature's shape. In many instances, the operational model relates certain elements that might not be accessible or too sophisticated for experimentation to pick up. In these situations, RSM investigates the issue and illustrates the relationship between the variables and the response by presenting certain mathematical and statistical methods. Assume that response (Z) is a quality characteristic or some performance attribute of the system being controlled by factors X_1_, X_2_, …, X_n_. Eq. (1) illustrates the relationship between the controlling variables and the response, where ε denotes other sources of variables not taken into account in function f, a random variable assumed to have a normal distribution with a mean of zero and a variance of σ^2^. As a result, f function is the response function's expected value.

(1) Z = f (X_1_, X_2_, …,X_n_)+ε

Due to the f function's complexity, an experimentally based polynomial equation could be used to estimate it. If this function can be established, you can determine the variables' responses in areas where there is insufficient experimental evidence. Eq. (2) is an example of a second order polynomial function. According to Ref. [30], this model can be used to estimate an actual reaction surface surrounding any specified point (Eq. (2)).

(2) Z = a_0_ + a_1_X_1_ + a_2_X_2_ + … + a_n_X_n_ + a_12_X_1_X_2_ + a_13_X_1_X_3_ + … +a_1n_X_1_X_n_ + a_23_X_2_X_3_ +a_24_X_2_X_4_ + … +a_2n_X_2_X_n_ + … + a_(n-1)n_X_n-1_X_n_ + a_11_X^2^_1_ + … + a_nn_X^2^_n_

Jalali et al. [30] in 2023 provide a thorough explanation of the analysis of variance (ANOVA) procedure that should be used to determine the regression's significance. The occurrence of flood is projected for the points lacking historical data using RSM. Predicted data are produced by applying the historical data to the RSM analysis in the Design Expert 7 software. Additionally, through this analysis, the optimum flows without occurring floods in the Sefidrud River could be obtained.

### 2.2 Machine learning computation

Machine learning algorithms are utilised in this study to predict the occurrence of floods with high accuracy. Different algorithms are used to be compared by other indices to identify the most accurate method for flood estimation. 50 to 70 percent of the statistical data were used in the present study to develop a prediction model by RF, Bagging, SMOreg, MLP and ANFIS. The WEKA 3.9 programme is used to implement the modeling, and the results are validated using the remaining data to ensure that the prediction models are accurate. The primary goal is to track and estimate flood occurrence, compare the accuracy of various methods, and apply them to the DSS. The following explains some of the ML algorithms employed in the present study [31]. Furthermore, it should be noted that for the SMOreg, MLP, and RF models, 60 % of the data was allocated for training purposes. For the Bagging approach, this proportion was set at 50 %. The remaining data in each case was reserved for testing the models' performance.

#### 2.2.1 Random forest

A supervised machine learning approach called RF uses decision trees to handle non-linear regression and classification issues. Number decision trees constructed from various subsets of training data make up RFs. The class with the most votes become the prediction outcome for the model from each individual tree in the random forest. RFs operate via four key phases, choosing n randomly chosen samples from the dataset with k samples, building unique decision trees for every sample, each decision tree produces a result, to construct the final product, regression issues are averaged and categorisation issues are decided by majority vote. The strength against overfitting, ease of use and interpretation, suitability for a variety of data types, including regression, classification, and survival analysis, and the absence of any distributional assumptions regarding the predictor or response variables are just a few benefits that RFs offer. However, compared to similar methods, it takes longer because of the large number of decision trees [31].

#### 2.2.2 Multilayer perceptron (MLP)

A feedforward artificial neural network (ANN) with the ability to build sophisticated nonlinear models, the MLP is perfect for forecasting flood occurrence. An MLP has an input layer, a hidden layer, and an output layer, which together make up at least three nodes. Except for the input nodes, each node is a neuron with a nonlinear activation function. During training, MLP employs the supervised learning method known as backpropagation. The multiple layers and nonlinear activation of MLP set it apart from a linear perceptron [32]. The MLP begins when the network receives the relevant input parameters. These input parameters generate input signals, which are transmitted across the network starting from the input layer and moving via the hidden layer and output layer. Neurons in the input layer scale the input vector, which is multiplied by weights, a real-number variable. This information, including bias, is summarised by the neuron in the hidden layer (Eq. (3)) [33].

(3) $$y_{0}=\sum_{i=1}^{n}w_{i}x_{i}+b$$

The weighted sum of the data is still shown linearly at this point. The non-linearity occurs when the data is transferred through the transfer function (Eq. (4)) [33].

(4) $$f\left(x\right)=\frac{1}{1+e^{-x}}\;\text{and}\;y_{0}=f\left(\sum_{i=1}^{n}w_{i}x_{i}+b\right)$$

where y_0_ denotes the output, w_i_ and x_i_ denote the weight vector and scaled input vector, respectively, b stands for bias, f for transfer function, and x is the total sum of weighted inputs.

The accurate estimation of the number of neurons in the hidden layer is one of the most crucial steps in putting this strategy into practice. If there are too few or too many of these neurons, under-fitting and over-fitting, respectively, will take place. Finding the ideal number of neurons, which can be done through trial and error, is crucial. Islam and Murase [34] suggest the number of neurons in the hidden layer is not higher than twice the number of inputs.

#### 2.2.3 Adaptive neuro-fuzzy inference system

A sort of artificial neural network created by Ref. [35] is an adaptive neuro-fuzzy inference system, often referred to as ANFIS. Takagi-Sugeno type fuzzy inference systems are trained using a hybrid of least-squares and backpropagation gradient descent methods, which are used to determine the appropriate parameters. In order to create the fixed input-output pairings, it can be utilised to create a set of fuzzy “if-then” rules with the required membership functions. The ANFIS is a simple structure with a strong learning algorithm and fast processing. Because neural network backpropagation reduces the search space dimensions, a hybrid approach has the advantage of converging more quickly [36,37].

**Fig. 2:**
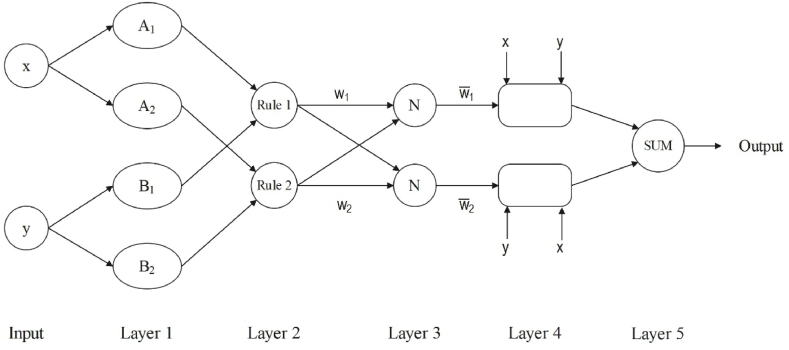
The overview of ANFIS method.

The ANFIS algorithm's structure is shown in Fig. 2, where x and y are the inputs to layer 1 and the ${O_{i}}^{1}$ refers to the node i's output. Below is a brief description of the algorithm process (Eqs. (5), (6))) [[36], [37], [38]].

(5) $$\begin{array}{l} {O_{i}}^{1}=\mu_{A_{i}}\left(x\right),\forall i\in \left\{1,2\right\}\\ {O_{i}}^{1}=\mu_{B_{i}}\left(y\right),\forall i\in \left\{1,2\right\} \end{array}$$

(6) $$\mu \left(x\right)=e^{-\frac{1}{2}{\left(\frac{x-\rho_{i}}{\alpha_{i}}\right)}^{2}}$$

where ${O_{i}}^{1}$ is the ith output of layer 1, μ represents the generalised Gaussian membership function, the A_i_ and B_i_ indicate the membership values of μ, the α_i_ and ρ_i_ are the premise parameter set. In the second and third layers the process is as follows (Eq. (7)) [36,37].

(7) $$\begin{array}{l} {O_{i}}^{2}=\mu_{A_{i}}\left(x\right)\times \mu_{B_{i}}\left(y\right)\\ {O_{i}}^{3}={\bar{w}}_{i}=\frac{\omega_{i}}{\sum_{i=1}^{2}\omega_{i}} \end{array}$$

in which w_i_ represents the ith output from the second layer.

The output of the third layer is multiplied with the Sugeno fuzzy rule's function in the fourth layer, as shown in Eq. (8) [36,37].

(8) $${O_{i}}^{4}={\bar{w}}_{i}f_{i}={\bar{w}}_{i}\left(p_{i}x+q_{i}x+r_{i}\right);\;\forall i\in \left\{1,2\right\}$$

in which p_i_, q_i_ and r_i_ are the resulting parameters of node i.

In order to determine the final result, the fifth layer uses the weighted average summation approach (Eq. (9)) [36,37].

(9) $${O_{i}}^{5}=\frac{\sum_{i}\omega_{i}f_{i}}{\sum_{i}\omega_{i}}$$

### 2.3 Case study

In this context, we initially delineate the characteristics of the Sefidrud River within our case study. Subsequently, we examine the topography of its catchment area. Finally, we analyze the meteorological and hydrological aspects of the catchment.

#### 2.3.1 The Sefidrud river characteristics

**Fig. 3:**
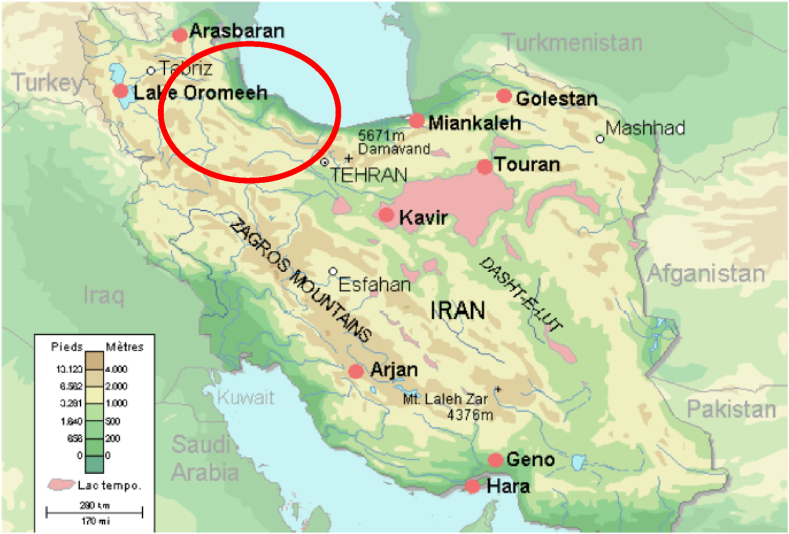
Location of SefidRud catchment shown on Iran's map.

**Fig. 4:**
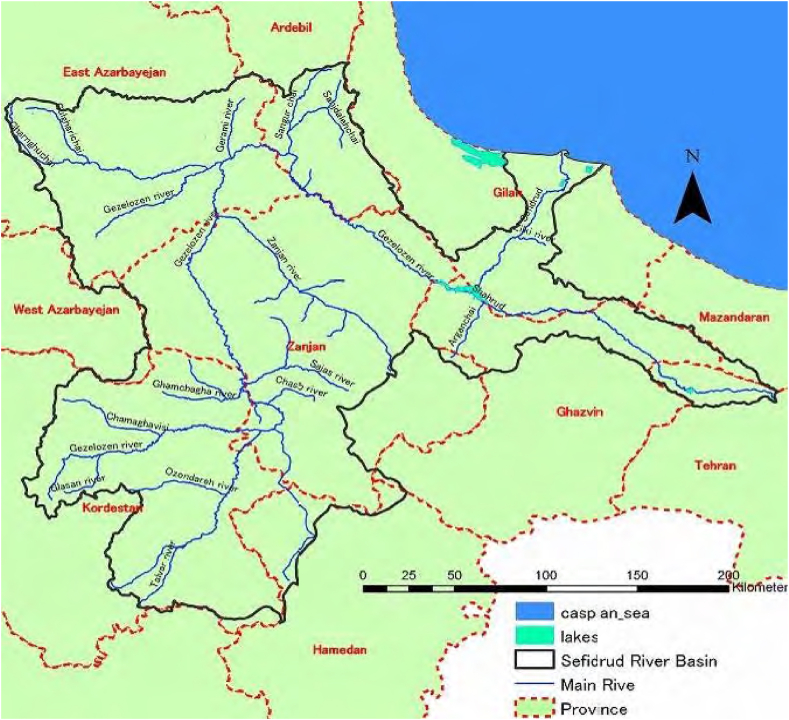
Border of SefidRud catchment and main tributaries forming two main headwaters of Shahrud and Gezelozen, which meet in the city of Manjil, creating one of the longest rivers in Iran, SefidRud.

The study focuses on the Sefidrud River basin, located in Iran, a region that is highly susceptible to flooding due to its unique geographical and climatic conditions. The Sefidrud River, one of the longest rivers in Iran, originates from the Alborz Mountains and flows into the Caspian Sea, traversing various topographical features, including mountainous regions and plains. These areas experience a cold, alpine climate with substantial snowfall during the winter months, leading to a significant snowmelt contribution to the river's flow in the spring. Also, the plains experience a more temperate climate with distinct wet and dry seasons. The wet season, typically from October to April, is characterized by heavy rainfall that often results in flooding. The Sefidrud River basin is highly vulnerable to flooding due to The combination of steep mountainous regions and low-lying plains creates conditions conducive to rapid runoff and accumulation of floodwaters, the seasonal patterns of heavy rainfall and snowmelt contribute to frequent flooding events, and, changes in land use, including deforestation and urban expansion, exacerbate flood risks by reducing natural water absorption and increasing surface runoff. The Sefidrud River originates from the confluence of Gezelozen and Shahrud at Manjil, in the North of Iran, Gilan province (Fig. 3, Fig. 4) and thereafter runs down the Alborz Mountain ranges up to Sefidrud delta and the Caspian Sea, 75 km away. The total size of its catchment is about 59,000 km^2^, which comprises the sub-basins and rivers as indicated in Table 1.

**Table 1:** Characteristics of rivers and main tributaries forming SefidRud river.

No	River	Catchment Area (km^2^)	River length (km)	Average Slop (1/I)
1	Shahrud River	4,850	210	1/110
2	Gezelozen River	48,600	670	1/340
3	Zanjan River	4,690	150	1/140
4	Talvar River	5,920	160	1/290
5	Sefidrud	59,090	750	1/360

**Fig. 5:**
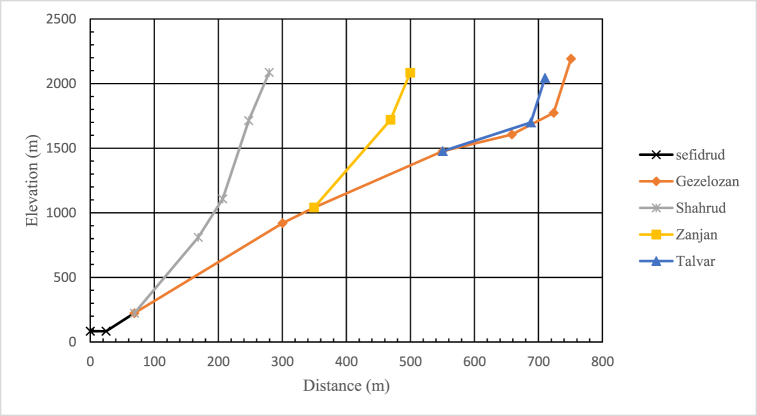
Mean elevation profile of the main rivers flowing into SefidRud.

Zanjan and Talvar river flow into the Gezelozen before reaching the watersmeet. Fig. 5 presents mean elevation profile of these rivers.

Average annual water yield of Sefidrud is approximately 4.8 billion cubic meters of which based on hydrometric data 4.4 billion comes from Gezelozen branch and 1.2 billion from Shahrud, collectively 5.6 billion cubic meters; the difference is due to water use in the upstream of the confluence. There is a considerable fluctuation between high and low flows in these rivers, i.e., between 0.5 and 2000 cubic meters per second for Gezelozen and 2.4 and 800 for Shahrud.

#### 2.3.2 Sefidrud catchment's topography

Most of the basin is located between the Alborz and the Zagros Mountain ranges and Gilan is the only Province situated north of the Alborz Mountains, south of the Caspian Sea in this basin. Mountains and hills are mostly observed in the study area. Alluvial plains, river alluvial plains, which is the most suitable aquifer, are distributed only in the Sefidrud delta at the Caspian sea's estuary. The next considerable aquifer are the terrace deposits that includes sand and gravel with clay.

#### 2.3.3 Sefidrud catchment's meteorology and hydrology

The annual average precipitation in the northern section of the Sefidrud River Basin is more than 1,000 mm whereas it varies from 200 mm to 400 mm in the southern part. Beyond 90 % of the average annual precipitation during the seven months between November and May in the south and between September and March in the North; although pluvial period for the latter, variation of a precipitation pattern does not appear.

The annual average evaporation in the southern part of Alborz range is four times of the precipitation, say, 2000 mm, while in the northern the value is less than 1500 mm. It is noteworthy that the evaporation measured by the pans is at most 70 % higher than the natural conditions.

The annual average temperature varies between −5 and 25 degrees of Celsius. Further, generally, it is cooler in the north than the southern part. The lowest and highest temperatures mostly occur in February and August respectively. Based on observatory stations in Loshan and Gilvan Peak flows start to occur in February and reach their peaks in April. Annual mean runoff ratio is estimated to be 0.22. That said, analysis of results shows that the runoff ratio decreases to a much lower value, around 0.09 during the dry years.

### 2.4 Statistical and hydrological analysis

In the first place, after acquisition of 51-year historical daily average hydrometric data, annual maxima for SefidRud river were extracted. In the region of the study, about 30 km upstream of two rivers of Gezelozen and Shahrud confluence and also to a reasonable extent downstream of the point on a straight reach at SefidRud river, there are hydrometric stations which have been installed and operated for the whole range of the study time. Therefore, these data only have been checked for the consistency of the data in these three different stations and there was no insufficiency or missing data throughout these years. The data series, before being used for the analysis, should pass stationary tests and clearance of outliers.

A change detection test of Man-Kendall for monotonic trends was applied. This method was originally put forward by Ref. [39] and was developed by Kendall [40]. This test has the advantage of not assuming a particular form of distribution for the observed data. For a time series of $X_{t}$ where t = 1,2, …, N; the Mann-Kendall statistic is calculated using Eq. (10) as follows [41]:

(10) $$S=\sum_{i=1}^{N-1}\sum_{j=i+1}^{N}\text{sgn}\left(X_{j}-X_{i}\right)$$

Sgn is the sign function and S has a null expected value E[S] = 0 and its variance is given by Eq. (11) [41]:

(11) $$Var\left[S\right]=\frac{1}{18}\left[N\left(N-1\right)\left(2N+5\right)-\sum_{m=1}^{M}t_{m}\left(t_{m}-1\right)\left(2t_{m}+5\right)\right]$$

in the above equation, m indicates the number of tied groups and t_m_ is the size of mth group. Z, as standardised statistics of test, following a standard normal distribution, is calculated as follows (Eq. (12)) [41]:

(12) $$Z=\left\{ \begin{array}{c} \frac{S-1}{\sqrt{Var\left[S\right]}}\;for\;S>0\\ 0\;for\;S=0\\ \frac{S+1}{\sqrt{Var\left[S\right]}}\;for\;S<0\; \end{array}\right.$$

**Fig. 6:**
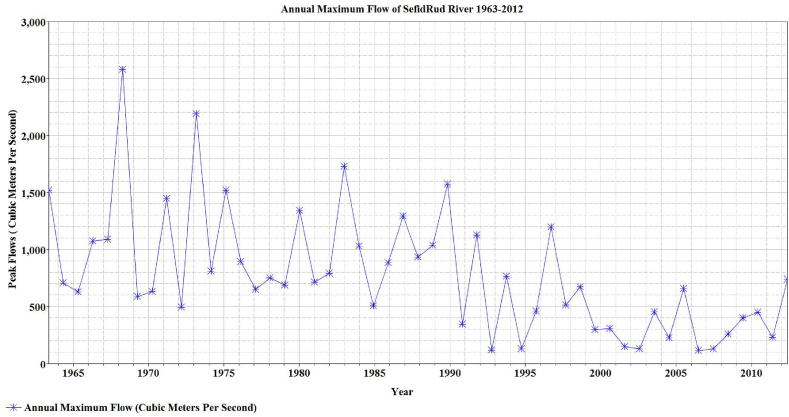
Time series of annual maximum of flows for SefidRud river from 1963 to 2012.

The null hypothesis H_0_ for Man-Kendall is: “no trend in hydrologic series”. If H_1_ is alternative hypothesis without specification of the increasing or decreasing nature of the monotonic trend, the hypothesis test is two-tailed and H_0_ would be rejected on the condition: $\left|Z\right|>Z_{1-\alpha /2}$ where α denotes the significance level. For the data of this study, and significance level of 0.05, Z was equal to −4.04 for S = −499 and Var(s) of 1.5E04. Considering $Z_{1-\alpha /2}$ = 2.24, the null hypothesis is rejected and the flood time series contains a trend. In Fig. 6, records of annual maximum have been presented which gives a holistic view of the variation of maximum annual floods over the course of time.

In order to have an exceedance probability, flood frequency analysis was conducted on these data using RStudio programming software. Generally, FFA is used for extrapolation by fitting a probability distribution function to existing data. Regarding the abovementioned Man-Kendall test's results the data series shows a descending trend and common distribution fitting models cannot be employed without temporal configuration. Coles (2001) [42] introducing nonstationary Generalised Extreme Value (GEV) distributions, expresses some sort of impossibility for deduction of a general theory of nonstationary extreme values except in a few limited cases. The underlying idea is to model distributions' parameters as a function of time. The main three components of the FFA are data choice, model choice and parameter estimator [43]. There are uncertainties in utilizing FFA which are summarised in Table 2 [44].

**Table 2:** Uncertainties involved in the application of FFA.

Uncertainty type	Sensitive to
1) Natural uncertainty	Nonstationary conditions
2) Model uncertainty	Choice of model
3) Parameter uncertainty	Fitting technique; goodness-of-fit test
4) Data uncertainty	Data choice, accuracy of observed/gauged data
5) Operational uncertainty	Human errors/decisions

The whole effort is to manage these uncertainties and minimise them. In river measurements, due to the wide range of discharges to be measured and complex nature of flow there are always some discrepancies between estimations and observations. For the Sefidrud River the measurement is ongoing for more than a half a century using propeller, and a reliable stage-discharge has been developed. A reasonable effort has been made to minimise data and operational uncertainties. The measurement is done in 0.2, 0.6 and 0.8 m of the depth from the water surface while section divisions on average are 2 m laterally. Moreover, the reach chosen during 60s are of the minimum change in planiform, geometry, and wide enough to mitigate secondary flows and vortices in order to minimise flow measurement inaccuracies.

Here, as there are limited choices due to nonstationary of the data, nonstationary GEV model has been adopted. One of its parameters should be estimated as function of time or a covariate. Therefore, it is assumed our random variable follows a GEV distribution while its parameters vary with time (Eq. (13)) [41]:

(13) $$X_{t}∼\text{GEV}\left(\beta \left(t\right).\alpha \left(t\right).\kappa \left(t\right)\right)$$

β(t).α(t).κ(t), are location, scale, and shape parameters, respectively. For modeling temporal change of mean of the model, considering linear dependence of location parameter β on time, Eq. (14) can be used to compute time dependent location parameter of GEV where $\beta_{1}$ indicates rate of change of the variable [41]:

(14) $$\beta \left(t\right)=\beta_{0}+\beta_{1}t$$

in this study, Eq. (15), has been plugged into the model although there are other types of relationships between time and location parameters such as polynomial or change point at a specific time of t_0_. There are other methods of introducing nonstationary into the model depending on temporal change of the component under consideration, i.e., variance or mean. In case of variance, scale parameter, α, can be used. It is noteworthy that the shape parameter, κ, as may lead to convergence instability, is seldom considered for trend modeling [41]. Finally, after the computation of model parameters, the value of a particular non-exceedance probability is computed using Eq. (15) [41]:

(15) $$x\left(F\right)=\beta +\frac{\alpha }{\kappa }\left[1-{\left(-\ln\;F\right)}^{\kappa }\right]$$

To calculate model parameters in this study, “ismev” package of RStudio programming tool for GEV modeling was used. By application of this method, $\beta_{0}$ = 916.64 with a standard error of 84.57, $\beta_{1}$ = −13.90 with a standard error of 2.473, α = 289.71 with a standard error of 38.485, and κ = −0.19 having a standard error 0.142. Results of flood frequency analysis using these parameters due to the assumption of linear descending trend do not yield reasonable results for the odd floods of having return periods of more than 100 years. Consequently, considering the fact that the results coming from nonstationary FFA may underestimate flood of this type, results of an ordinary FFA has been used for the sake of higher reliability to create a threshold for flood alarming system.

Since the early 1980s, the United States employed Bulletin 17B guidance for FFA, which uses Log Pearson Type III (LP3) probability distribution for annual peak flow estimation in unregulated streams [45].

**Fig. 7:**
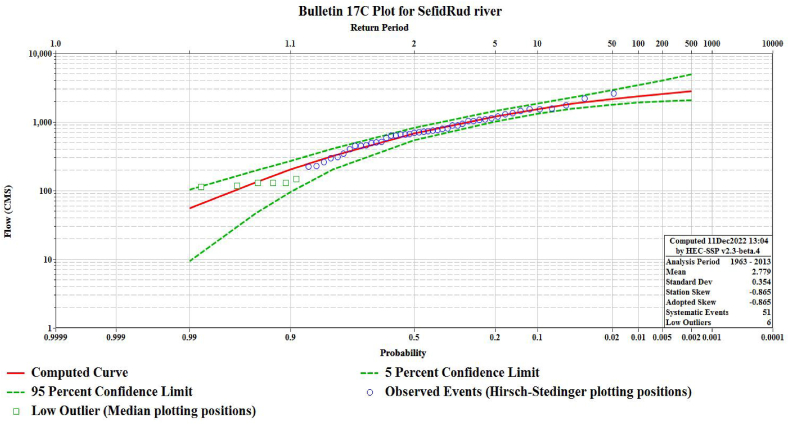
Diagram of exceedance probability estimated curve in the case study of present research.

It also introduces some adjustments for non-standard data i.e., missing years, flows below the gage base, low outliers and historical events (Eq. (16)) [46].

(16) $$f\left(x\right)=\left|\beta \right|{\left\{\beta \left[\ln\;x-\epsilon \right]\right\}}^{\alpha -1}\frac{\exp\left\{-\beta \left[\ln\;x-\epsilon \right]\right\}}{x\Gamma \left(\alpha \right)}$$

in the above equation, β, α and ϵ are scale, shape, and location parameters respectively. The most recent introduced procedure by the US geological survey (USGS) is the Bulletin 17c. In this research the FFA has been conducted following the aforementioned guidance so that the parameter estimation uncertainties become minimised. The bulletin 17c still employs Log Pearson Type III but instead of typical moment method for parameter estimation, uses Expected Moments Algorithms (EMA). This procedure also provides an improved confidence interval and has mechanism of outlier detection and exclusion while modeling the data. It introduces every peak flow within a range, whether observed or not, and the range can be replaced when a real observation exists. Fig. 7 and Table 3 show an estimated curve and result for Sefidrud river by bulletin 17c in HEC-SSP 2.3.

**Table 3:** Results of FFA using EMA and LP3 for estimation of exceedance probabilities and corresponding discharges.

Percent Chance Exceedance	Computed Curve flow in CMS	Variance Log (EMA)	Confidence Limits of Flow in CMS	
			0.05	0.95
0.2	2781.5	0.010	4895.9	2082.0
0.5	2564.8	0.007	4029.2	1998.7
1	2375.1	0.005	3450.6	1909.4
2	2159.8	0.004	2925.9	1786.9
5	1829.8	0.002	2292.1	1551.0
10	1539.5	0.002	1848.6	1303.7
20	1206.6	0.002	1431.7	1009.2
50	674.9	0.003	824.4	542.9
80	319.9	0.006	406.0	201.9
90	201.8	0.012	271.7	95.8
95	132.7	0.021	194.4	46.6
99	54.9	0.058	102.2	9.4

## 3 Results and discussion

For a thorough analysis, we begin by scrutinizing the outcomes of the RSM. Following that, we assess the results derived from the accuracy of various ML methods. Subsequently, we construct a DSS to facilitate an in-depth analysis for analysing the risk of flood in our case study.

### 3.1 The RSM outcomes

Based on the historical hydrological data of the studied rivers the RSM analysis has been implemented to find the flows where there is no data and also find the optimum flow in a way that no floods occur in the Sefidrud River. Using the flow data of the rivers and the time series in the Design Expert 7.0.0 software Eq. (17) is achieved.

(17) Flood occurance = +0.0579–0.0002 × A – 0.1216 × B – 0.0301 × C + 0.1174 × D + 0.00009 × AB + 0.00002 × AC – 0.00005 × AD – 0.000003 × BC – 0.0000006 × BD + 0.000002 × CD

As it is illustrated in Table 4, it is suggested that the model is a 2-factor interaction (2FI) equation and according to the Analysis of Variance (ANOVA), which is fully demonstrated in Table 5, the flow data in the Shahrud river with P-value<0.02 and F-value>5 is more important than other parameters. Also, the interaction of the GezelOzan and Sefidrud flow is considered significant in the flood occurrence.

**Table 4:** The outcomes of the statistical modelling through sensitivity analysis.

Source	Std. Dev.	R-Squared	Adjusted R-Squared	Predicted R-Squared	PRESS
Linear	0.3295	0.5593	0.5210	0.3131	7.7845
2FI	0.2289	0.8151	0.7688	0.3347	7.5400
Quadratic	0.2372	0.8213	0.7518	−0.1603	13.1502
Cubic	0.2467	0.9141	0.7315	−214.4450	2441.7100

**Table 5:** Interaction of variables in RSM analysis.

Source	Sum of Squares	Mean Square	F-Value	p-value Prob > F
A-Time	0.0001	0.0001	0.0016	0.9680
B-GezelOzan flow	0.1151	0.1151	2.1967	0.1461
C-Shahrood flow	0.3457	0.3457	6.5975	0.0141
D-Sefidrood flow	0.1080	0.1080	2.0607	0.1589
AB	0.1061	0.1061	2.0252	0.1625
AC	0.0154	0.0154	0.2935	0.5910
AD	0.0990	0.0990	1.8889	0.1770
BC	0.0076	0.0076	0.1456	0.7048
BD	0.4587	0.4587	8.7539	0.0052
CD	0.0059	0.0059	0.1125	0.7391
Residual	2.0958	0.0524		

**Fig. 8:**
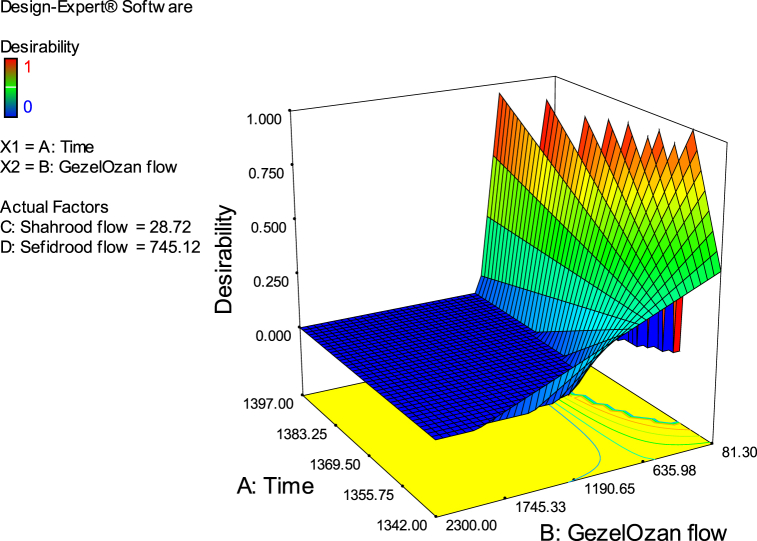
Desirability diagram of the RSM analysis.

Eq. (17) provides a 2FI equation that describes the flood occurrence in the Sefidrud river, as a function of certain features. Specifically, the equation includes flow of three rivers in addition to the time. This equation could also help optimise the flow in all rivers in a way that no flood occurs during the specific time period. By incorporating the operational features described in the equation, it can potentially predict the river flow in all three rivers, especially where the range of the data is missing, this could save time and resources that would otherwise be required to measure the data in the field. However, the desirability of the model is not the same in all ranges of the data. Fig. 8 illustrates the range that the model is more desirable which is when Gezelozan flow is less than 635 m^3^/s and the flow in the Shahrud river is around 28 m^3^/s.

**Fig. 9:**
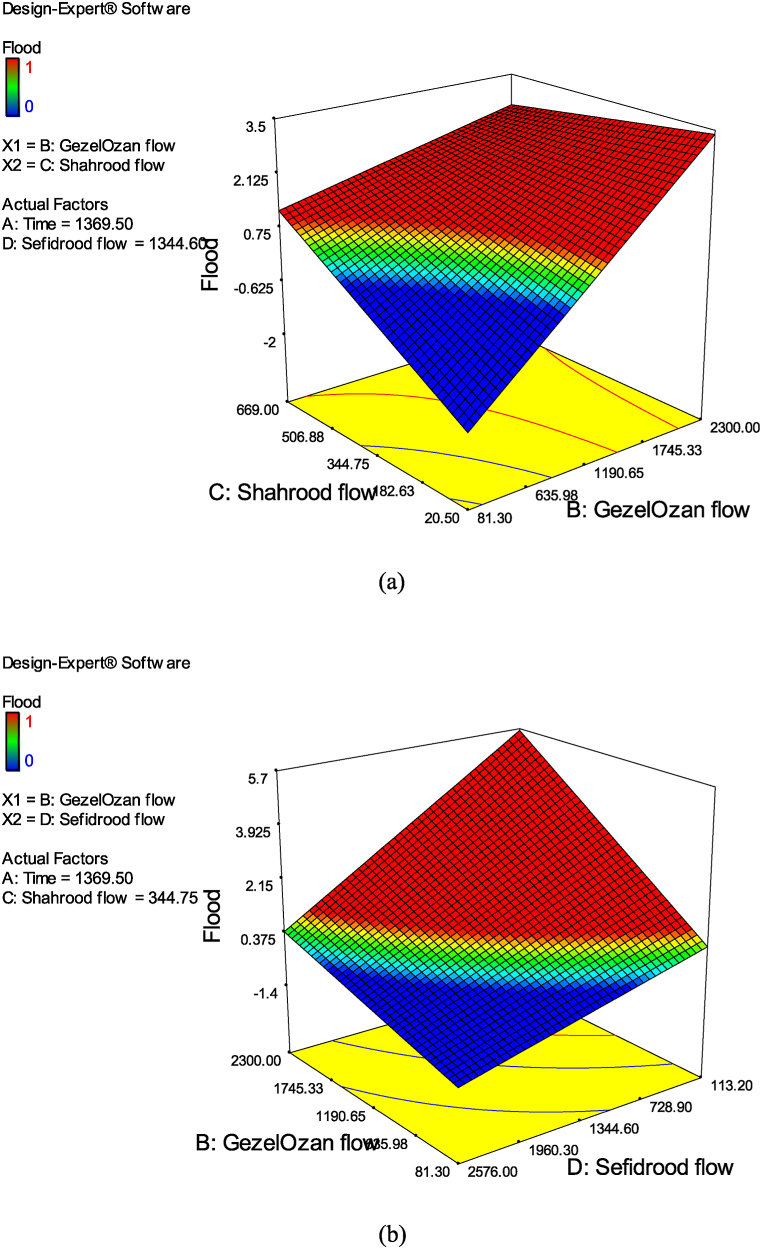

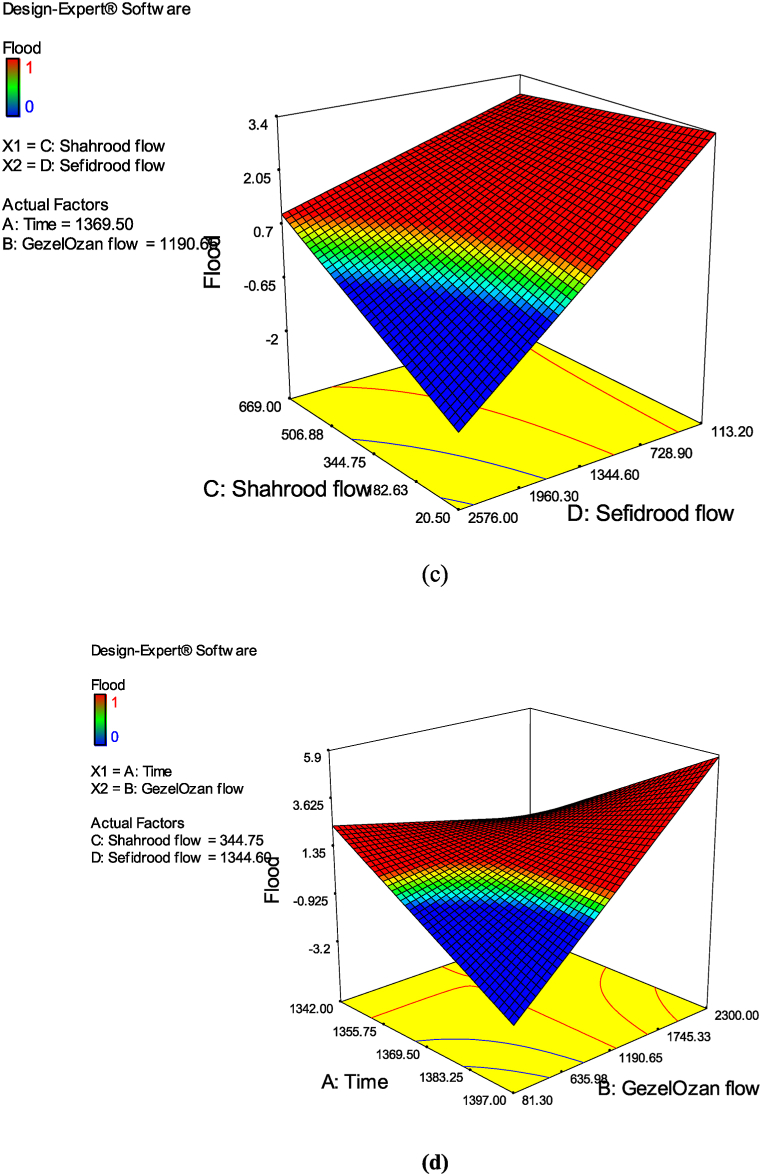
Outcomes of the RSM analysis (a–d).

The RSM provided valuable insights into the global dynamics of flood occurrences across the parameter space. The interaction between river flows, as an indicator of flood risk, appears hydrologically sound at a conceptual level. However, the model's goodness-of-fit, reflected by an R-squared value of 0.82, indicates potential for improvement. Additional influential factors such as rainfall, soil moisture, and drainage conditions could enhance the model's explanatory power and account for more of the variance in flood responses. Fig. 9 presents the results of a sensitivity analysis based on an ANOVA of the flood equation's parameters. The graphs depict the binary interactions of flow and time variables. Specifically, Fig. 9(a) illustrates the interaction between the Shahrood and GezelOzan flows. Fig. 9(b) shows the interaction between the GezelOzan and Sefidrood flows. Fig. 9(c) highlights the interaction between the Shahrood and Sefidrood flows. Finally, Fig. 9(d) focuses on the interaction between time factors and the GezelOzan flow, about flood occurrence.

From these plots, it is evident that flood occurrences are more strongly influenced by the flows in the Shahrood and GezelOzan rivers, a finding that aligns with expectations. Notably, the slope of the graph for the Shahrood River is steeper than the others, reinforcing earlier results that suggest a higher sensitivity of flood risk to the Shahrood flow. This heightened sensitivity underscores the critical role of Shahrood flow in flood prediction and management, confirming its significance in hydrological assessments of the region. In conclusion, while the interactions between river flows are key in explaining flood risk, the model could be further refined by integrating additional environmental factors, leading to a more comprehensive understanding of flood dynamics.

**Fig. 10:**
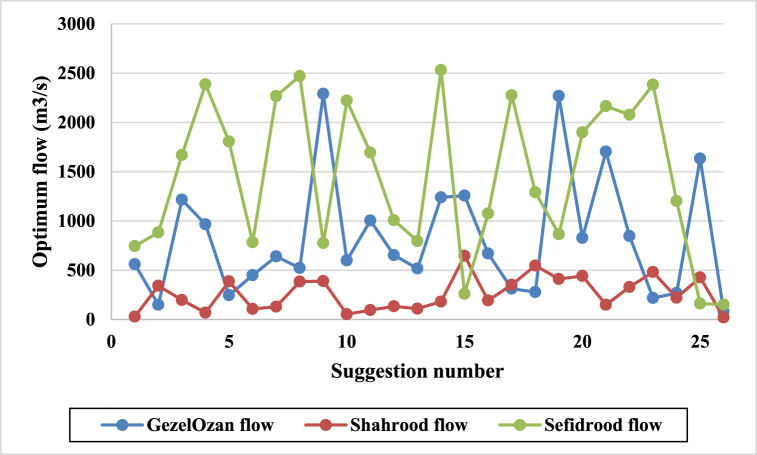
Optimum flow conditions in all the rivers without flood occurring.

Also, the results of the optimization process through RSM analysis are demonstrated in Fig. 10. This diagram suggests the amount of flow in three rivers in a way that no flood occurs during a year. As it is illustrated, the maximum flow in the Sefidrud River, which is the result of the conjunction of the other two rivers, is about 2500 m^3^/s. This is while the flow in the GezelOzan river is at most 1200 m^3^/s and the flow in the Shahrood river is below 400 m^3^/s. In the same condition, when the real flow in Sefidrood is about 2500 m^3^/s, flood has occurred and the flow in GezelOzan and Shahrood rivers is around 2300 and 700 m^3^/s, respectively.

### 3.2 ML results

The results obtained from machine learning algorithms are illustrated in Table 6. It compares four machine learning algorithms on the task of flood prediction for the Sefidrud River basin - SMOreg, MLP, Bagging and RF. Several evaluation metrics are reported to assess model performance: correlation coefficient, mean absolute error, root mean squared error, relative absolute error and root relative squared error.

**Table 6:** Outcomes of ML prediction.

Parameters of ML algorithm	SMOreg	MLP	Bagging	RF
Correlation coefficient	0.644	0.823	0.865	0.868
Mean absolute error	0332	0.143	0.097	0.104
Root mean squared error	0.446	0.312	0.250	0.261
Relative absolute error (%)	72.5 %	31.19 %	21.82 %	22.60 %
Root relative squared error (%)	88.87 %	62.23 %	51.83 %	51.97 %

Looking at the results, RF achieves the best performance overall, with the highest correlation to actual values at 0.868 and the lowest errors across all metrics. Its ensemble modeling approach with many decision trees can effectively capture the nonlinear patterns in flood occurrence. MLP comes second in performance, with correlation at 0.823 and errors lower than the other algorithms except RF. Its neural network structure provides nonlinearity that suits the complex flood prediction task reasonably well. Bagging beats only SMOreg, demonstrating some benefits from model ensembling compared to a single model. SMOreg performs the worst among the four, with linear regression limitations making it insufficiently flexible for this problem.

For more perspective, flood estimating systems typically target correlations of at least 0.8 and root mean squared errors below 50 % of the average flow. The achieved metrics meet and exceed these common accuracy guidelines. Specifically for the Sefidrud, the average annual flow is around 580 m^3^/s based on the data. The RF with RMSE of 0.261 m^3^/s, therefore, represents just 0.04 % of the average flow. This is well within the acceptable error threshold of 50 % for operational estimating systems. We can further analyze required accuracy levels by looking at flood severity categories. Minor floods may be defined as flows between the 1–2 year return period, major floods between 2 and 10 years, and extreme floods above 10 years. For minor flood estimation on the Sefidrud, the RMS error would need to be constrained within 10–15 % of the 2-year return flow of around 2000 m^3^/s. The achieved 0.261 m^3^/s meets this requirement. For major and extreme floods, errors within 20–25 % could be reasonable. Again, the RF model meets these accuracy needs for high flow predictions.

The results of ML indicators in all four applied algorithms are demonstrated in Table 7. In binary classification, metrics are computed separately for c0 and c1 to provide a balanced and detailed evaluation of the model's performance. This approach helps in understanding how well the model identifies true positives and negatives, manages false positives, and balances precision and recall for each class. It is crucial for handling class imbalance, gaining detailed insights, and guiding targeted performance improvements. Thus, reporting metrics separately for both classes ensure a comprehensive understanding of the model's effectiveness and areas for enhancement [47].

**Table 7:** Outputs of ML indicators in the present study.

Algorithms	TP Rate	FP Rate	Precision	Recall	F-measure	MCC	ROC Area	PRC Area	Class
SMOreg	0.92	0.533	0.742	0.92	0.821	0.448	0.693	0.733	C0
	0.467	0.08	0.778	0.467	0.583	0.448	0.693	0.563	C1
	0.75	0.363	0.755	0.75	0.732	0.448	0.693	0.669	Weighted Avg.
MLP	0.88	0.267	0.846	0.88	0.863	0.623	0.816	0.793	C0
	0.733	0.12	0.786	0.733	0.759	0.623	0.816	0.767	C1
	0.825	0.212	0.823	0.825	0.824	0.623	0.816	0.784	Weighted Avg.
Meta. Bagging	0.813	0.278	0.839	0.813	0.825	0.529	0.829	0.88	C0
	0.722	0.188	0.684	0.722	0.703	0.529	0.829	0.714	C1
	0.78	0.245	0.783	0.78	0.781	0.529	0.829	0.82	Weighted Avg.
RF	0.92	0.4	0.793	0.92	0.825	0.564	0.848	0.877	C0
	0.6	0.08	0.818	0.6	0.692	0.564	0.848	0.773	C1
	0.8	0.28	0.803	0.8	0.792	0.564	0.848	0.838	Weighted Avg.

The provided metrics evaluate the performance of the SMOreg algorithm in both training and testing phases as per Table 7. The metrics for classes c0 and c1, along with the weighted averages, shed light on the algorithm's overall effectiveness and areas for improvement. During the training phase, the algorithm demonstrates strong performance, particularly for class c0. This is evident from the high True Positive Rate (TP Rate) and Recall of 0.920, indicating that the algorithm correctly identifies 92 % of the positive instances of c0. The Precision for c0 is also substantial at 0.742, meaning a significant proportion of the predicted positives are accurate. These metrics culminate in an F-Measure of 0.821, suggesting a well-balanced precision-recall trade-off. However, the False Positive Rate (FP Rate) of 0.533 for c0 indicates that over half of the actual negatives are misclassified as positives, highlighting an area for improvement. For class c1, the training phase reveals some challenges. The TP Rate and Recall are notably lower at 0.467, meaning the algorithm identifies less than half of the actual positives correctly. Despite this, the Precision for c1 is high at 0.778, indicating that when the algorithm predicts a positive, it is correct most of the time. The F-Measure for c1 is 0.583, reflecting the lower recall. The FP Rate for c1 is low at 0.080, showing the algorithm does a good job at not misclassifying actual negatives as positives. The Matthews Correlation Coefficient (MCC) for both classes is the same at 0.448, suggesting a moderate correlation between the predicted and actual classes. When considering the testing performance, the weighted averages provide a holistic view. The weighted TP Rate and Recall are both 0.750, indicating that the model correctly identifies 75 % of the instances across both classes. The weighted Precision is slightly higher at 0.755, showing that a substantial proportion of the predicted positives are true positives. The F-Measure is 0.732, reflecting a balanced performance in terms of precision and recall. The overall FP Rate is 0.363, which is relatively high and suggests some room for reducing false positives. The ROC Area, which measures the algorithm's ability to distinguish between classes, is 0.693 for both classes and the weighted average, indicating a moderate level of discrimination. The Precision-Recall Curve (PRC) Area further provides insights into the performance with respect to the minority class. With a PRC Area of 0.733 for c0 and 0.563 for c1, the algorithm shows better precision-recall balance for c0. The weighted PRC Area of 0.669 indicates an overall moderate performance. In conclusion, the SMOreg algorithm performs robustly during the training phase, particularly for class c0, but encounters challenges with class c1 in terms of recall. The testing phase metrics, represented by the weighted averages, reveal that while the model maintains reasonable precision and recall, it faces issues with a higher false positive rate. The ROC and PRC Areas suggest moderate discriminatory power and precision-recall balance. These insights highlight the strengths of the algorithm in predicting positives accurately while indicating areas such as false positive reduction and recall improvement for c1 that need attention for enhanced performance.

The MLP algorithm demonstrates robust performance across both classes, as reflected in the provided metrics in Table 7. For class c0, the TP Rate and Recall are both 0.880, indicating that the model correctly identifies 88 % of the positive instances. The Precision for class c0 is 0.846, showing that the majority of predicted positives are accurate. This results in a high F-Measure of 0.863, signifying a good balance between precision and recall. The FP Rate for class c0 is 0.267, which indicates that around 26.7 % of actual negatives are incorrectly classified as positives. For class c1, the TP Rate and Recall are slightly lower at 0.733, meaning the model correctly identifies 73.3 % of the positive instances. However, the Precision for class c1 is 0.786, indicating that the predicted positives are mostly correct. This results in an F-Measure of 0.759. The FP Rate for class c1 is 0.120, which is relatively low. The MCC for both classes is 0.623, suggesting a strong correlation between the predicted and actual classes. The weighted average metrics, with a TP Rate and Recall of 0.825 and Precision of 0.823, indicate balanced overall performance. The weighted F-Measure is 0.824, reflecting the model's consistency. Additionally, the ROC Area of 0.816 indicates a high ability to distinguish between classes, and the PRC Area of 0.784 suggests a strong precision-recall balance. Overall, the MLP algorithm shows high accuracy and reliability in classifying both classes, with room for improvement in reducing the false positive rate for class c0.

The Bagging algorithm shows solid performance across both classes, as indicated by the metrics provided in Table 7. For class c0, the TP Rate and Recall are both 0.813, signifying that the algorithm correctly identifies 81.3 % of the positive instances. The Precision for class c0 is 0.839, indicating that the majority of predicted positives are accurate. This results in an F-Measure of 0.825, reflecting a good balance between precision and recall. However, the FP Rate for class c0 is 0.278, suggesting that 27.8 % of actual negatives are misclassified as positives. For class c1, the TP Rate and Recall are 0.722, meaning the algorithm correctly identifies 72.2 % of the positive instances. The Precision for class c1 is 0.684, indicating that a significant portion of predicted positives are correct, though there is room for improvement. The F-Measure for class c1 is 0.703, showing a reasonable balance between precision and recall. The FP Rate for class c1 is 0.188, which is relatively moderate. The MCC for both classes is 0.529, suggesting a moderate correlation between the predicted and actual classes. The weighted average metrics present a balanced overall performance, with a TP Rate and Recall of 0.780 and a Precision of 0.783. The weighted F-Measure is 0.781, indicating the algorithm's consistency across different classes. Moreover, the ROC Area of 0.829 indicates a high ability to distinguish between the classes, and the PRC Area of 0.820 suggests a strong precision-recall balance. Generally, the Bagging algorithm performs well in classifying both classes, showing high accuracy and reliability, with opportunities for improvement in reducing the false positive rate, particularly for class c0.

The RF algorithm exhibits strong performance across both classes (Table 7). For class c0, it achieves a high TP Rate and Recall of 0.920, indicating the algorithm correctly identifies 92 % of positive instances. The Precision for class c0 is 0.793, showing that a large proportion of predicted positives are correct. This results in an F-Measure of 0.852, reflecting a robust balance between precision and recall. However, the FP Rate for class c0 is 0.400, suggesting that 40 % of actual negatives are misclassified as positives. For class c1, the TP Rate and Recall are 0.600, meaning the algorithm correctly identifies 60 % of the positive instances. The Precision for class c1 is 0.818, indicating that most of the predicted positives are accurate. The F-Measure for class c1 is 0.692, demonstrating a reasonable balance between precision and recall. The FP Rate for class c1 is low at 0.080, showing the algorithm does well in not misclassifying actual negatives as positives. The MCC for both classes is 0.564, suggesting a moderate correlation between the predicted and actual classes. The weighted average metrics reveal a balanced overall performance, with a TP Rate and Recall of 0.800 and Precision of 0.803. The weighted F-Measure is 0.792, indicating consistency in the algorithm's performance across different classes. Furthermore, the ROC Area of 0.848 indicates a strong ability to distinguish between classes, and the PRC Area of 0.838 suggests a good precision-recall balance. Overall, the Random Forest algorithm performs well, showing high accuracy and reliability in classifying both classes. However, there is room for improvement in reducing the false positive rate for class c0 and enhancing the recall for class c1.

The superior performance of nonlinear machine learning models like RF and MLP illustrates their suitability for the complex flood prediction task compared to traditional linear regression approaches. The ability to model nonlinear relationships allows them to capture better the intricacies of how different factors like rainfall, soil moisture, drainage and antecedent river flow affect flood occurrence. In particular, the ensemble strategy of RF provides an advantage over a single model by reducing variance and avoiding overfitting. The multiple decision trees essentially vote on the prediction outcome, improving robustness. MLP also generalises well with multiple hidden layers, extracting hierarchical feature representations from the data.

The study results align with recent literature showing machine learning models outperforming conventional hydrologic techniques for tasks like rainfall-runoff modeling and streamflow estimation. While physical process-based models have an interpretability advantage, they struggle to match the predictive accuracy of data-driven methods when training data is sufficient. However, the high R-squared values also warrant some caution. The exceptional fits could stem from overfitting on noisy historical data. Testing on out-of-sample data would better validate true generalisation capability. Regularisation strategies and robust training procedures will be important to optimise model complexity and avoid overfitting.

**Fig. 11:**
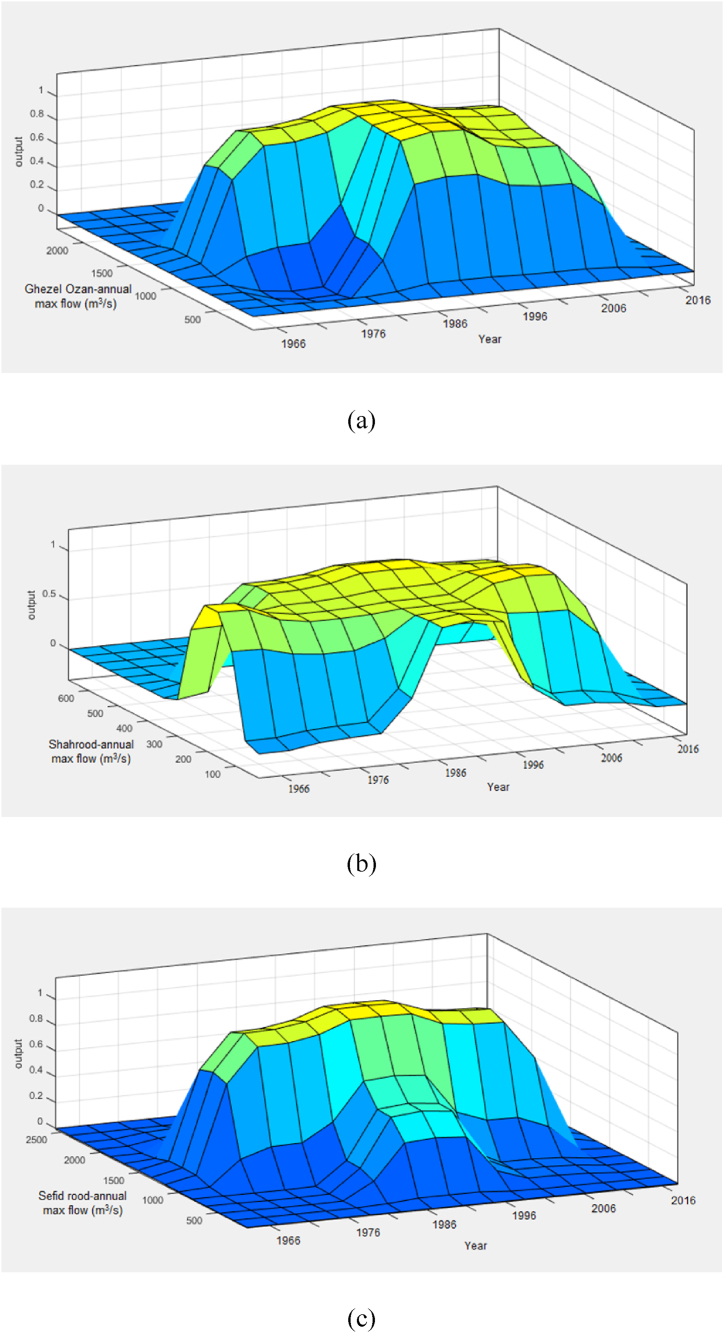

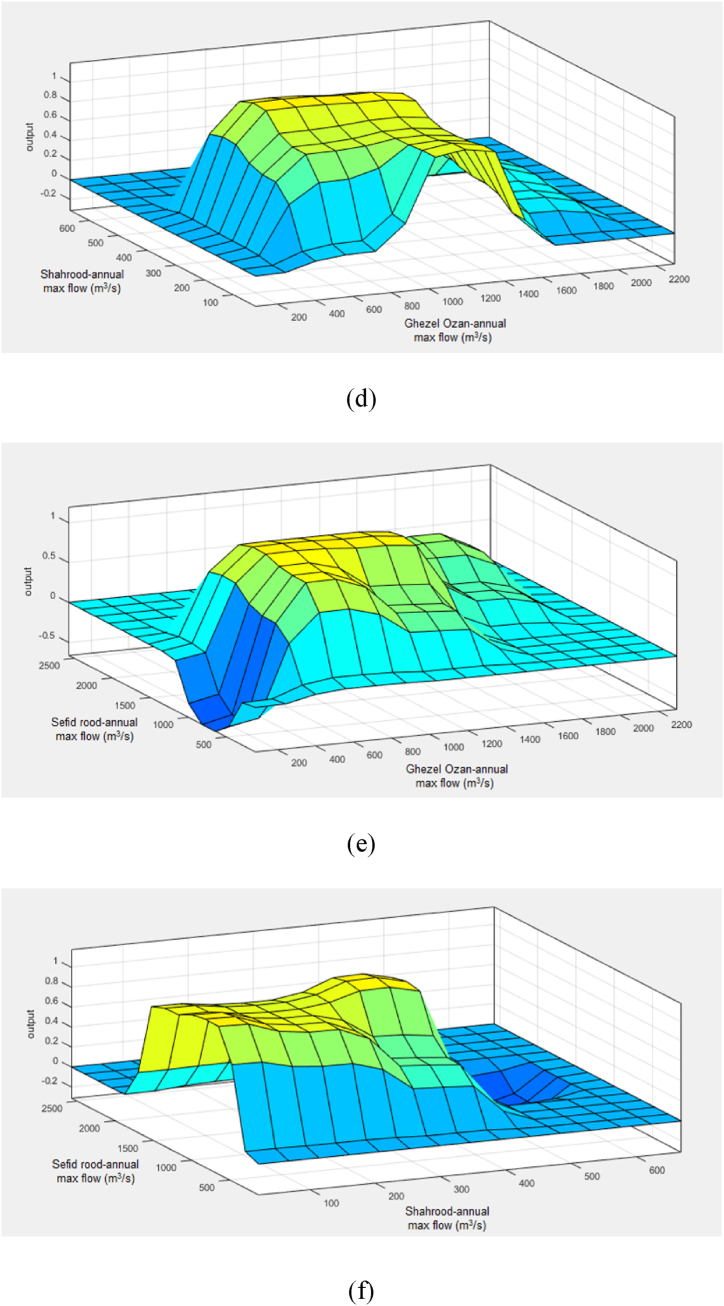
Flood prediction results of the ANFIS analysis; a) GhezelOzan river flow, b) Shahrood river flow, c) Sefidrood river flow, d) Shahrood vs. GhezalOzan flow, e) Sefidrood vs. GhezelOzan flow and, f) Sefidrood vs. Shahroos maximum flow in a year.

The results of the ANFIS analysis are presented in Fig. 11 and Table 8. Fig. 11 visualizes the maximum river flows within the planning horizon from 1966 to 2016 for the Gezelozan, Shahrood, and Sefidrood rivers, as well as comparisons between these rivers. Specifically, Fig. 11(a) illustrates the maximum flow of the Gezelozan River during this period. Fig. 11(b) depicts the maximum flow for the Shahrood River. Fig. 11(c) highlights the maximum flow of the Sefidrood River. Fig. 11(d) compares the maximum flows of the Shahrood and Gezelozan rivers. Fig. 11(e) compares the maximum flows of the Sefidrood and Gezelozan rivers. Fig. 11(f) compares the maximum flows of the Sefidrood and Shahrood rivers.

**Table 8:** Outcomes of ANFIS modelling.

Regression Statistics	
Multiple R	0.99
R Square	0.99
Adjusted R Square	0.99
Standard Error	0.000251

As shown in the figures, the ANFIS approach, applied to the dataset spanning from 1966 to 2016, demonstrates exceptional accuracy in predicting flood events, achieving an R-squared (R^2^) value of 0.99. This high R^2^ indicates a near-perfect fit, confirming the ANFIS model's effectiveness in capturing the complex relationships between river flows and flood occurrence, making it a powerful tool for flood forecasting in this region.

Achieving a very high R-squared value of 0.99 with ANFIS on historical data means the model fits the training data exceptionally well. There are a few potential reasons for getting such a high R-squared, the model has high complexity with many parameters to fit the data, ANFIS has several membership functions and rules that can be tweaked to fit historical data very closely. The flood prediction problem has strong underlying relationships between the inputs and outputs that ANFIS is able to capture well. The physical dynamics may be suitable for fuzzy modeling. Large amounts of quality training data containing accurate flood records and relevant inputs.

An R-squared of 0.99 on training data alone is very high, which could mean the model is overfit. Since ANFIS can emulate nonlinear functions, it's possible to overfit by matching the noise and irregularities in the data. However, the RSM analysis outcomes confirm the ANFIS results when they are compared together, which means overfitting is not the reason for the high accuracy of this approach.

According to the literature, Lawal et al. [48] created ML models using Decision Tree, Logistic Regression & Support Vector Classification to predict floods in Nigeria based on 33 years of rainfall data. Logistic Regression gave the most accurate results with high performance accuracy & recall. Decision Tree was reasonably good, but Support Vector Classification performed poorly with the small dataset. Sankaranarayanan et al. [49], compared deep neural network, SVM, K-Nearest Neighbors and Naive Bayes models for flood prediction based on temperature and rainfall data. The deep neural network with 0.95 precision and 91.18 % accuracy showed the highest accuracy for flood forecasting using monsoon parameters, outperforming the other machine learning models. Motta et al. [50] developed a flood prediction system using ML models, including Logistic Regression, SVM, Gaussian Naive Bayes, RF, KNN, and MLP with GIS techniques. The RF model performed the best with a Matthew's Correlation Coefficient of 0.77 and Accuracy of 0.96, providing an effective tool for urban management and resilience planning. ANFIS combines fuzzy logic and neural networks to adaptively learn from data, refining fuzzy rules and membership functions. In contrast, RF constructs an ensemble of decision trees through bootstrapping and feature randomization. Each algorithm may perform better depending on the nature of the learning data. ANFIS (our study) excels with data featuring complex, non-linear relationships where fuzzy logic can effectively model uncertainties and adapt to varying inputs [51].

Widiasari and Nugroho [52] used a wireless sensornNetwork for data collection and a MLP deep learning model for time series forecasting of water elevation based on rainfall and weir water level data. The system achieved a low mean absolute percentage error of 3.64 % error between predicted and actual water levels. MLP performed better than multiple linear regression for predicting downstream water elevation. In our study, ANFIS outperformed MLP. This difference could stem from variations in the data sources used. Widiasari and Nugroho [52] predicted water levels, whereas our study focused on assessing flood probabilities.

Won et al. [53] presented an urban flood forecasting and warning process using a rainfall-runoff model and deep learning for South Korea. The rainfall-runoff model was calibrated to an R^2^ of 0.63–0.79. Deep learning models like LSTM and Bidirectional LSTM were used for 10-min forecasting, with the bidirectional model achieving R^2^ of 0.9 for 30 min lead time. This aims to contribute to flood damage reduction in South Korean urban streams through accurate warnings. Both ANFIS and LSTM models leverage their adaptive nature to effectively learn from and make predictions or classifications based on diverse datasets, highlighting their versatility in various applications requiring nuanced pattern recognition and decision-making capabilities [54,55]. Therefore, both ANFIS (our study) and LSTM had appropriate results for flood prediction.

Kimura et al. [56], introduced transfer learning to a convolutional neural network (CNN) flood model to reduce computational costs instead of training with large datasets from scratch. Time series data was converted to images for the CNN. In the source domain, CNN time series classification had around 10 % error. With transfer learning in the target domain, mean error by 15 % compared to CNN without transfer learning. Additionally, CNNs, like ANFIS (as studied in our research), are neuro-based machine learning models. It can be inferred that neural networks may yield superior results compared to other machine learning approaches for high-fluctuation events such as floods.

Sahoo et al. [57], employed an ANFIS model and integrated with meta-heuristic Grey Wolf Optimization and Grasshopper Optimization Algorithm for flood prediction in River Mahanadi, India. They reached the maximum amount of RMSE equal to 0.02 for all the measurements and found that ANFIS-Grasshopper Optimization Algorithm model outperforms the other approaches. Indra and Duraipandian [58] proposed a framework with two modes - data visualisation and data analysis - for forecasting floods accurately using location features. It utilises techniques like levy flight K-means clustering, Gaussian Kernel-ANFIS for visualisation, and conjugate gradient deep neural network for analysis. Experiments showed that the framework achieved 96.66 % prediction accuracy, outperforming existing methods. Tabbussum and Dar [59] used two ANFIS algorithms with Takagi-Sugeno fuzzy inference systems that were explored for flood forecasting, trained using hybrid and backpropagation methods. The hybrid-trained ANFIS model with 12 inputs achieved the best performance, with mean square error of 0.00034, R^2^ of 97.066 %, RMSE of 0.018, Nash–Sutcliffe model efficiency of 0.968, MAE of 0.0073 and combined accuracy of 0.018, showing its potential for flood prediction. Comparative studies, such as that by Ref. [60], developed a hybrid ANFIS-Genetic Algorithm (GA) model to predict river flow in Turkey's Seyhan River, comparing it with classical ANFIS, ANN, and LSTM models. The ANFIS-GA model outperformed other methods, achieving the highest R^2^ values (≈0.9409 and ≈ 0.9263) for two flow measurement stations, demonstrating its effectiveness in river flow prediction. However, to fully contextualize these results, it would be beneficial to compare them with studies specific to the Sefidrud River basin or similar Iranian watersheds. The findings of [60] support the results of our research based on the R^2^ statistical indicator. They utilised a metaheuristic algorithm to reduce errors in ANFIS. However, in our study, we discovered that by adjusting certain features such as the number and types of membership functions, we can enhance the performance of ANFIS without requiring extensive computational resources like GA. For instance, Dodangeh et al. [61] applied machine learning models to flood susceptibility mapping in northern Iran, providing a relevant regional comparison. They integrated resampling algorithms (random subsampling and bootstrapping) with machine learning models (generalised additive model-GAM, boosted regression tree-BT, multivariate adaptive regression splines-MARS) to improve flood susceptibility predictions in Ardabil Province, Iran. The bootstrapped GAM model (BT-GAM) performed best, achieving an receiver operating characteristics curve of 0.98, true skill statistic of 0.93, and correlation coefficient of 0.91, outperforming standalone models and other machine learning techniques. In our study, we achieved ANFIS model results exceeding an R^2^ value of 0.95.

### 3.3 Decision support system

**Fig. 12:**
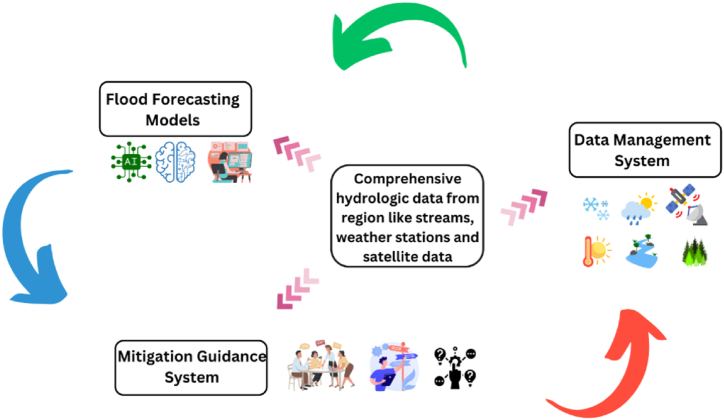
Schematic model of DSS provided to manage flash flood.

The flood prediction models and hydrologic analysis will be integrated into a DSS to guide flood mitigation actions in the Sefidrud River basin. The DSS will have three main components, which is also demonstrated in Fig. 12.

#### 3.3.1 Data management system

This will consolidate and quality control hydrologic data from sources like stream gauges, weather stations and satellite data. Database management capabilities will enable systematic storage and retrieval of data to feed models.

#### 3.3.2 Flood estimation models

This will house the developed ANFIS model along with other statistical and hydrologic models to generate flood estimation and probabilities. Ensemble modeling will also be utilised to improve predictions. Automated workflows will run models in real-time or at specified intervals.

#### 3.3.3 Mitigation guidance system

This will leverage model outputs to provide actionable risk-based recommendations to decision makers. Expert knowledge will be encoded into logic rules and procedures for flood warning escalation protocols, reservoir operations, evacuation planning, etc. User interfaces will provide clear visualisations and interpretability.

The flexible DSS architecture will allow integration of new data sources, models and business logic rules over time. Outputs will be accessible via interactive dashboards for relevant stakeholders. Operators can initiate model runs and simulate various scenarios to support decisions. The system will also have GIS capabilities for spatial analyses and maps.

The initial implementation will focus on retrospective simulation and testing. The eventual vision is usage for real-time estimation and warning to enhance flood preparedness and response. Collaboration with emergency managers, community leaders and other end-users will be critical throughout development to maximise usability and adoption. Overall, the DSS can provide an adaptive platform to support integrated flood management in the Sefidrud River basin.

According to the literature review, various connections exist between MCDM models and ML algorithms in the implementation of DSS. Some studies focus on spatial data assessment for decision-making [15], while others concentrate on hydrological data analysis [7,10]. In spatial data analysis, integrating ML models with geographical tools can identify high-risk zones. By applying MCDM techniques, appropriate decisions can be made for each zone under different scenarios. This approach allows for a nuanced assessment that considers various risk factors and outcomes, leading to more informed and effective decision-making.

On the other hand, DSS implementations based on hydrological data [7,10] utilize ML algorithms to analyze the possibility of flooding. This analysis involves threshold appraisal to manage alarms and provide early warnings. When the likelihood of flooding increases, the system can make real-time decisions to mitigate risks. This real-time capability is crucial for managing emergencies and reducing potential damage. The primary distinction between these two types of DSS lies in the timing of decision-making. Hydrological data based DSS can make quicker decisions compared to those based on spatial data, due to the immediate nature of the data and the urgent need for timely responses in flood situations.

**Fig. 13:**
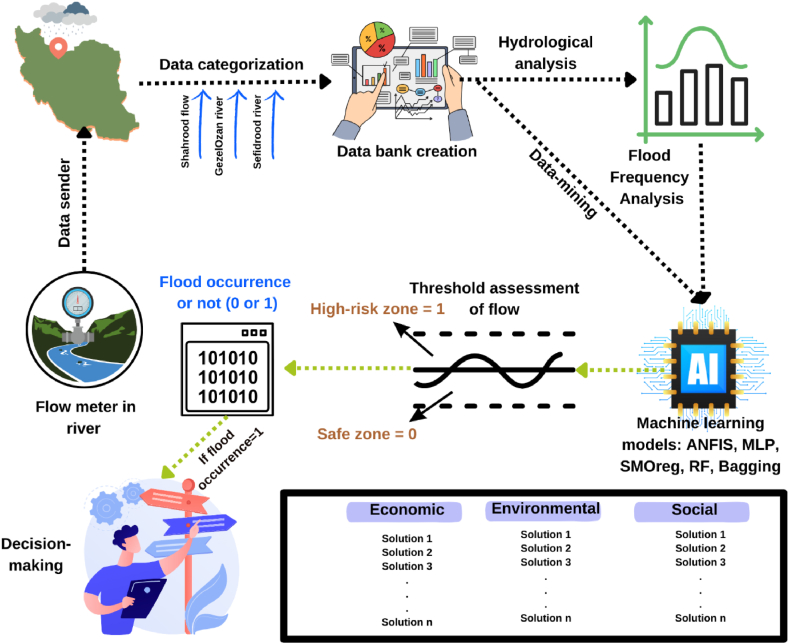
The schematic plan of DSS model in the present study.

The present study focuses on a DSS based on hydrological data. It aims to develop a model, as illustrated in Fig. 13, that can effectively predict flood risks and facilitate real-time decision-making to enhance disaster management and response strategies.

In the present study, the flow data from three rivers is first analyzed using both ML algorithms and flood frequency analysis. The impact of different flood flows is examined to understand the behavior and characteristics of each river during flood events. Subsequently, flood flow is evaluated against established flood thresholds based on historical data. This evaluation helps predict the likelihood of flood occurrence, allowing for prioritization of decision-making processes. The specific decisions and their prioritization will be the focus of the subsequent study. To ensure comprehensive decision-making, various MCDM models are considered. These models will be evaluated in alignment with the Sustainable Development Goals (SDGs), incorporating economic, environmental, and social criteria. By integrating these criteria, the study aims to develop solutions that are not only effective in flood management but also sustainable and beneficial to the broader community. This holistic approach ensures that the decisions made are well-rounded and contribute to long-term resilience and sustainability.

## 4 Conclusions and future works

This study demonstrates the significant potential of ML algorithms for improving flood estimation accuracy, with broader implications for global flood risk management. Through the development and evaluation of multiple ML and ANN algorithms, utilizing historical hydrometric data from the Sefidrud and its tributaries, the research uncovers valuable insights. The results showcase the superiority of state-of-the-art nonlinear models, specifically RF and MLP, over traditional linear regression approaches for complex flood prediction tasks. The RF model, in particular, achieves a remarkable correlation coefficient of 0.868 and demonstrates low errors with an RMSE of just 0.261 m^3^/s, equivalent to 0.04 % of the average annual flow. The ANFIS model also delivers highly accurate predictions with a correlation coefficient of 0.99, meeting the typical accuracy requirements for operational flood estimation systems. This analysis not only emphasizes the superior performance of modern ML methods but also provides valuable insights into algorithm selection for flood estimation.

The methodologies and findings are not confined to the Sefidrud River basin but are applicable to various geographical regions facing similar hydrological challenges. By showcasing the superior performance of advanced ML models such as RF, MLP, and ANFIS, this research provides a benchmark for other regions to adopt and customize these techniques based on their specific data and requirements. The integration of such data-driven models into DSS can significantly enhance real-time flood estimation and disaster management strategies, thereby mitigating the adverse impacts of floods globally.

While the study demonstrates promising accuracy on historical data, translating these findings into robust real-world prediction systems requires additional research. Recommendations for future work include expanding model input parameters beyond river flows to encompass other hydrologic and meteorologic variables such as rainfall, soil moisture, and snowpack levels. Integrating satellite and remote sensing data, where available, can provide a broader spatial perspective. Testing more complex neural network architectures, like LSTM networks, can model longer temporal patterns effectively. Rigorous out-of-sample validation on unseen data, uncertainty quantification methods, and setting up operational forecasting systems integrated with disaster management agencies are vital steps to enhance real-time applications.

This study acknowledges limitations, emphasizing the need for further research. Future endeavours should incorporate additional input parameters, and satellite data, and explore more complex neural network architectures for longer temporal patterns. Rigorous out-of-sample validation, uncertainty quantification methods, and operational forecasting system setups are essential for real-world applicability. Collaborating with local hydrologists and emergency planners is recommended to refine predictions and co-develop actionable risk-based mitigation guidance. Expanding the data-driven modelling approach to other flood-prone regions and conditions will contribute to evaluating generalizability.

In conclusion, pursuing these research directions will translate promising results into robust and trustworthy artificial intelligence systems. This, in turn, enhances disaster resilience and contributes to effective flood risk reduction strategies. The study's contributions lie in not only showcasing the accuracy of ML models but also in providing a roadmap for future research and practical implementations in the domain of flood forecasting and risk management.

## Data availability statement

Data will be made available on request.

## CRediT authorship contribution statement

**Ali S. Chafjiri:** Writing – original draft, Validation, Software, Resources, Methodology, Investigation, Formal analysis, Conceptualization. **Mohammad Gheibi:** Writing – original draft, Visualization, Validation, Resources, Methodology. **Benyamin Chahkandi:** Writing – original draft, Visualization, Software, Resources, Project administration, Methodology. **Hamid Eghbalian:** Writing – original draft, Methodology, Formal analysis, Data curation. **Stanislaw Waclawek:** Writing – original draft, Visualization, Supervision, Project administration. **Amir M. Fathollahi-Fard:** Writing – review & editing, Writing – original draft, Visualization, Methodology, Conceptualization. **Kourosh Behzadian:** Writing – original draft, Visualization, Software, Conceptualization.

## Declaration of competing interest

The authors declare the following financial interests/personal relationships which may be considered as potential competing interests: The corresponding author, Prof. Amir M. Fathollahi-Fard, is an Associate Editor in Information Science for Heliyon and was not involved in the editorial review or the decision to publish this article. If there are other authors, they declare that they have no known competing financial interests or personal relationships that could have appeared to influence the work reported in this paper.
